# Physical Activity and Cardiorespiratory Fitness Are Beneficial for White Matter in Low-Fit Older Adults

**DOI:** 10.1371/journal.pone.0107413

**Published:** 2014-09-17

**Authors:** Agnieszka Zofia Burzynska, Laura Chaddock-Heyman, Michelle W. Voss, Chelsea N. Wong, Neha P. Gothe, Erin A. Olson, Anya Knecht, Andrew Lewis, Jim M. Monti, Gillian E. Cooke, Thomas R. Wojcicki, Jason Fanning, Hyondo David Chung, Elisabeth Awick, Edward McAuley, Arthur F. Kramer

**Affiliations:** 1 The Beckman Institute for Advanced Science and Technology, University of Illinois, Urbana, Illinois, United States of America; 2 Department of Psychology, University of Iowa, Iowa City, Iowa, United States of America; 3 Department of Kinesiology and Community Health, University of Illinois, Urbana, Illinois, United States of America; Max Planck Institute for Human Cognitive and Brain Sciences, Germany

## Abstract

Physical activity (PA) and cardiorespiratory fitness (CRF) are associated with better cognitive function in late life, but the neural correlates for these relationships are unclear. To study these correlates, we examined the association of both PA and CRF with measures of white matter (WM) integrity in 88 healthy low-fit adults (age 60–78). Using accelerometry, we objectively measured sedentary behavior, light PA, and moderate to vigorous PA (MV-PA) over a week. We showed that greater MV-PA was related to lower volume of WM lesions. The association between PA and WM microstructural integrity (measured with diffusion tensor imaging) was region-specific: light PA was related to temporal WM, while sedentary behavior was associated with lower integrity in the parahippocampal WM. Our findings highlight that engaging in PA of various intensity in parallel with avoiding sedentariness are important in maintaining WM health in older age, supporting public health recommendations that emphasize the importance of active lifestyle.

## Introduction

Participation in physical activity and high levels of cardiorespiratory fitness (CRF) have protective effects on brain structure and function and are associated with later onset or lower degree of age-related cognitive decline [1], [2]. Disruption of axons and myelin in white matter (WM) is considered one of the primary mechanisms underlying age-related cognitive decline [3], [4]. Therefore, maintaining WM structural connectivity may be one of the key factors for preserving brain function and high cognitive performance necessary for independent living in old age. However, little is known about the associations of different levels of physical activity (PA), sedentary behavior, and CRF with the integrity of aging WM.

Much of the research examining PA and brain health has relied on subjective assessments of PA. Such self-reports, in contrast to quantitative measures such as accelerometry, fail to provide objective and accurate estimates of non-exercise lifestyle activities such as moderate PA (climbing stairs), light PA (housework, gardening) and sedentary behavior (prolonged periods of sitting with little movement, such as watching TV [5]). Importantly, different levels of PA are associated with different physiological mechanisms and benefits; for example, general sedentariness may even negate the benefits of sporadic MV-PA [6], [7]. Surprisingly, objective measures of PA using accelerometry have never been examined in association with brain health in older adults. Thus, the independent effects of sedentariness, PA, and CRF are on brain health remain unclear. Epidemiological studies using objective measures of energy expenditure have shown that greater overall PA is related to lower incident of dementia [8]. In adults who do not exercise, which is true for most older adults, it is low intensity PA, not MV-PA, that accounts for most energy expenditure [9]. In addition, more time spent on light PA means less sedentary behavior, which has strong adverse effects on the cardiovascular system, metabolism, and is related to higher mortality [10]. These findings highlight the necessity of objectively measuring PA to tease apart physiological mechanisms associated with PA of different intensity levels, which may differentially relate to the brain health. Finally, although MV-PA is more efficient for increasing CRF than light PA [11], studies using objective measures of PA revealed that middle and older age women who meet the guidelines for engagement in limited MV-PA do not spend less time in sedentary behavior than their low-active peers [6]. Therefore, adults engaging in MV-PA may still experience some negative physiological effects of sedentary behavior [7], which may obscure the associations between CRF and brain health measures. Clearly, some aspects of lifestyle behaviors that influence brain aging cannot be captured by the CRF measure alone. CRF typically measured objectively as maximum oxygen consumption is a sum of oxygen “supply factors” (pulmonary diffusion capacity, cardiac output, erythrocyte levels, and capillary density in muscles) and muscle mitochondrial respiration rate [12]–[14]. Although CRF can be increased by PA, genetic factors also contribute to CRF that can boost or limit activity-induced gains [15]. Thus, in order to understand the independent effects of physical activity (sedentariness, light PA, MV-PA) and CRF on WM integrity in aging, different intensities of PA need to be objectively assessed, in addition to CRF.

Magnetic resonance imaging enables the non-invasive assessment of integrity of aging WM. Age is the primary predictor of the appearance of lesions called WM hyperintensities (WMH) on T2-weighted images [16]. The pathogenesis of WMH is complex and includes occlusion of small cerebral vessels, local ischemic changes, and damage to the blood-brain barrier with chronic leakage of ventricular and blood plasma fluid into the WM [17], [18]. Second, age-related degeneration of WM microstructure can be captured as decreased fractional anisotropy (FA) measured with diffusion tensor imaging (DTI). FA is a measure of the directional dependence of diffusion [19], and reflects fiber density, integrity, and coherence within a voxel [20]. Reduced FA in aging has been linked to loss of axon and myelin integrity [21], [22]. Thus far, only four studies have investigated the relation between CRF and WM structure in healthy older adults. Three relatively small cross-sectional studies including a large fraction of very high fit older adults showed a positive relationship between CRF and FA in the anterior body corpus callosum ([23]; n = 26), anterior cingulum ([24]; n = 15), and single clusters in the inferior and superior longitudinal fasciculi ([25]; n = 20). A one-year aerobic exercise intervention study showed that increases in CRF were positively related to increases in general frontal and temporal lobe FA ([26]; n = 70). In addition, [25] showed that life-long engagement in MV-PA was linked to 83% reduction in deep WMH volume in Master's athletes compared to low-active controls. Although not representative of a typical low-active aging population, these preliminary studies on samples with very fit older adults are important by showing there may be a positive relationship between CRF and WM microstructure in anterior corpus callosum, cingulum, fronto-parietal connections and temporal WM, as well as associations between life-long history of MV-PA and WMH volume in healthy older individuals.

Our study goes beyond the above studies by combining objective measures of PA and CRF with two MRI measures of WM health (FA and WMH volume) in a large group of typically low–fit healthy adults (n = 88, age 60–78, 33 males). PA was objectively measured with an accelerometer worn on the hip for at least 10 waking hours for 7 days and was classified into sedentary behavior, light PA, and MV-PA [5]. We assessed CRF as the maximum oxygen consumption during a graded maximal exercise test. Unlike previous studies (e.g. [23]), we did not exclude participants with WMH but instead quantified the lesion volume on T2-weighted images as a marker of age-related WM damage. This made our sample more generalizable to a normal aging population and allowed inclusion of participants older than 70, given that only 4% of adults above age of 65 do not present WMH [27].

Our study had two main aims. First, we aimed to test whether in healthy low-active and low-fit older adults CRF conjointly with and PA is related to WM health, measured by combining FA with WMH volume. We predicted that, in general, greater CRF and PA would be related to higher WM integrity. The second aim, given that our first hypothesis is true, was to investigate in more detail the multiple interacting pathways linking PA to WM health. Here, our main goal was to identify in post-hoc exploratory analyses the relationships between different levels of PA intensity and WM health in older adults, and to test the role of CRF in these relationships. We predicted that greater PA would be related to lower WMH volume [25] and higher FA in five regions. These included two regions within the temporal lobe: parahippocampal WM and core temporal WM containing the inferior longitudinal fasciculus, given that exercise interventions and higher CRF showed beneficial effects on temporal regions and the hippocampus [28]–[33]. In addition, we revisited previous findings by measuring FA in three frontal regions that related to greater CRF: anterior corpus callosum, dorsal anterior cingulum, and superior longitudinal fasciculi [23]–[25]. With regard to PA intensity, we considered two alternative hypotheses. Better WM health could be related to greater MV-PA, as MV-PA is most efficient in increasing CRF and inducing CRF-related protective effects on the brain. Alternatively, in older population, higher levels of light PA or less sedentary behavior could act on WM via higher overall energy expenditure and reducing risks related to sedentariness. In addition, although FA and WMH are often related in the posterior periventricular regions, in other regions they probe different aspects of WM health and are not redundant [22], [34]. As outlined above, the same holds for PA and CRF. Thus, we hypothesized there may be a dissociation of the effects of CRF and PA on WMH and FA in healthy aging.

Our findings confirmed our expectations that better physical health was related to WM health. In particular, greater PA and lower sedentariness were associated with lower WMH volume and higher FA, especially in the temporal WM regions, but our data did not support a significant relationship between CRF and WM health in the frontal regions.

## Methods

### Participants

We recruited 103 community-dwelling healthy, older adults (33 males). The sample contained more females because less older males met the inclusion criteria or showed willingness to participate in the study. Eligible participants met the following criteria: (1) were between the ages of 60 and 79 years old, (2) were free from psychiatric and neurological illness and had no history of stroke or transient ischemic attack, (3) scored ≥23 on the Mini-Mental State Exam (MMSE) and >21 on a Telephone Interview of Cognitive Status (TICS-M) questionnaire, (4) scored <10 on the geriatric depression scale (GDS-15), (5) scored ≥75% right-handedness on the Edinburgh Handedness Questionnaire, (6) demonstrated normal or corrected-to-normal vision of at least 20/40 and no color blindness, (7) cleared for suitability in the MRI environment; that is, no metallic implants that could interfere with the magnetic field or cause injury, no claustrophobia, and no history of head trauma. The participants were a pre-intervention cross-sectional subsample from an on-going randomized controlled exercise trial (“Influence of Fitness on Brain and Cognition II” at ClinicalTrials.gov, clinical study identifier NCT01472744), from whom good quality DTI and T2 data (as described in the following sections), as well as CRF and PA data were available. We furthermore excluded participants who had MMSE score <27, in order to limit the analyses to cognitively healthy older adults and exclude those with possible mild cognitive impairment. Seventy-two participants (83%) of the sample reported no participation in regular PA (maximum of two moderate bouts of PA/week) in the past six months. The remaining 17% reported engaging in some exercise upon recruitment; however, the subsequent accelerometer data analysis revealed that as many as 40% of them did not met the minimum recommendations for PA (>150 min of moderate PA per week). Similarly, fourteen (19%) of the self-reported “low-active” adults met the criteria of >150 min of moderate PA per week. This highlights the necessity of objective assessment of PA for accurate sample description, in addition to self-reports. In total, based on the accelerometer data, only 26% (n = 23, 9 women) of the 88 participants met the minimum criteria for PA for older adults and only 3 of them met the recommended criteria based on vigorous activity (>60 min/week; [35]; one was subsequently excluded due to high CRF, see Section 2.3). In sum, we define our sample as low-fit and low-active although capable of performing exercise (i.e. no physical disability that prohibits mobility).

The 88 participants had mean body mass index (BMI) M_BMI_  = 30±6 kg/m^2^, systolic blood pressure M_sysBP_  = 131±13 mm Hg and diastolic blood pressure M_diasBP_  = 81±7 mm Hg. Out of 88 participants, 69 (78%) were normotensive, while 18 (22%) were hypertensive, defined as either systolic BP >140 mmHg or diastolic BP >90 mm Hg. Twenty participants (23%) had normal BMI, while 68 (77%) were overweight (BMI >25). If stratified based on BMI or BP, the groups did not differ on any of the WM or fitness variables of interest, and therefore BMI and BP were not included as in the following analyses.

### Physical activity assessment

Participants were instructed to wear the GT3× ActiGraph accelerometer (ActiGraph; Pensacola, Florida) for seven consecutive days on an elastic belt on the left hip during all waking hours, except for when bathing or swimming. The participants completed a daily log to record the time that the accelerometer was worn, and this log was used to verify the accelerometer data for processing with the ActiLife v5.6.0 software. For the purposes of this study, a valid day of data consisted of at least 10 hours of valid wear-time, with a valid hour defined as no more than 30 consecutive minutes of zero counts with one minute sampling epochs (Table 1). Only data for individuals with a minimum of three valid days of wear time were included in analyses [36]. Based on this criterion, two females were excluded from analyses. The remaining 86 participants (28 males) had on average 6.8±0.8 valid days of measurement (range 3–8), resulting in 90.7% of the sample having 6 or more valid days required to reliably measure sedentary behavior [36].

**Table 1:** Variables of interest: descriptive statistics and correlations with age.

Variable	n	Mean±SD*	Range*	*r* with age**	p-value
*Age (years)*	88	65±4	60–78	–	–
*Education (years)*	86	16±3	11–26	−0.21	0.059
*CRF* (mL/kg/min) ***					
Males	28	26±7	12–43	**−0.47**	0.012
Females	55	20±4	12–31	−0.06	0.661
					
*Physical activity*					
*(hours/day)*					
Sedentary	86	8.9±1.3	5.8–11.6	0.08	0.489
Light	86	4.6±1.2	2.3–7.6	0.01	0.938
MV-PA	86	0.27±0.25	0.01–1.22	**−0.26**	0.016
*Aceelerometer valid hours/day*	86	13.7±1.3	11.4–19.4	0.05	0.652
*WMH volume (voxels)*	86	813±1233	1–7290	**0.25**	0.021
*Fractional anisotropy (FA)*					
Anterior cc	86	.67±.03	.59–.75	−**0.22**	0.046
Anterior cingulum	86	.47±.03	.40–.54	**−0.27**	0.012
Superior longitud. fasci.	86	.46±.03	.41–.52	**−0.24**	0.025
Parahippocampal WM	81	.55±.03	.49–.62	**−0.39**	<.001
Temporal lobe WM	81	34±.03	.27–.42	−0.15	0.172

*Raw data.

** WMH and MV-PA were ln-transformed for correlations with age and valid hours were winsorized.

*** The values are after excluding two outliers >2.5 SD.

Each valid measurement epoch was classified into sedentary, light, moderate, and vigorous physical activity based on displacement magnitude and frequency. We used activity intensity cut-off ranges appropriate for older adults [5] using MeterPlus v4.2 software (Santech, Inc.; San Diego, CA). Sedentary behavior was defined as <100, light activity as 100–1951, moderate activity as 1952–5723, and vigorous activity as >5724 counts/epoch. The total epochs (i.e. minutes) of each intensity, divided by total valid days, yielded average time spent daily in a specific physical activity intensity (note that in Table 1 we further divided these variables by 60 to express the time in hours). Only nine participants showed any vigorous activity during the measurement week. We therefore summed moderate and vigorous activity to obtain a “moderate-to-vigorous activity” (MV-PA) variable [37]. Observed MV-PA was positively skewed and we performed a natural log-transformation of this variable for further analyses. The daily valid hours of two participants were >2.5SD and were winsorized with respect to the distribution of the whole sample before being entered into correlations and regressions.

### Cardiorespiratory fitness assessment

All participants obtained physician's approval to engage in cardiorespiratory fitness (CRF) testing. CRF was defined as peak oxygen consumption [ml/kg/min], measured with indirect calorimetry during a modified Balke graded maximal exercise test on a motor-driven treadmill test. Oxygen consumption (VO_2_) was calculated from expired air sampled at 30-s intervals until peak VO_2_ was reached or the test was terminated due to volitional exhaustion and/or symptom limitation. CRF was defined as the highest recorded VO_2_ value (VO_2_max) after two of three criteria were met: (1) a plateau in VO_2_ after increase in workload; (2) a respiratory exchange ratio >1.10, and (3) a maximal heart rate within 10 bpm of their age-predicted maximum. Our subjects represented a broad range of CRF values, with extremely high values for 2 female participants (>±2.5 SD and beyond the 90% peak VO_2_max percentile (superiorly/excellent fit) according to gender- and age-specific norms; ACSM's Guidelines for Exercise Testing and Prescription, www.acsm.org. Accessed 2014 June 12). They were therefore considered outliers in our largely low-fit sample and their values were removed from further analyses. As one female participant did not complete the treadmill test, the final sample for CRF assessment was 83 (28 males).

### MRI acquisition

Diffusion-weighted and high in-plane resolution T2-weighted images (for WMH volume estimation) were acquired on a 3T Siemens Trio Tim system with 45 mT/m gradients and 200 T/m/sec slew rates (Siemens, Erlangen, Germany). All images were obtained parallel to the anterior-posterior commissure plane with no interslice gap. T2-weighted images consisted of 35 4-mm-thick slices with an in-plane resolution of 0.86×0.86 (256×256 matrix, TR/TE  = 2400/63 ms, FA  = 120). DTI images were acquired with a twice-refocused spin echo single-shot Echo Planar Imaging sequence [38] to minimize eddy current-induced image distortions. The protocol consisted of a set of 30 non-collinear diffusion-weighted acquisitions with b-value  = 1000 s/mm^2^ and two T2-weighted b-value  = 0 s/mm^2^ acquisitions, repeated two times (TR/TE  = 5500/98 ms, 128×128 matrix, 1.7×1.7 mm^2^ in-plane resolution, FA  = 90, GRAPPA acceleration factor 2, and bandwidth of 1698 Hz/Px, comprising 40 3-mm-thick slices).

### Assessment of White Matter Hyperintensity (WMH) volume

**Figure 1:**
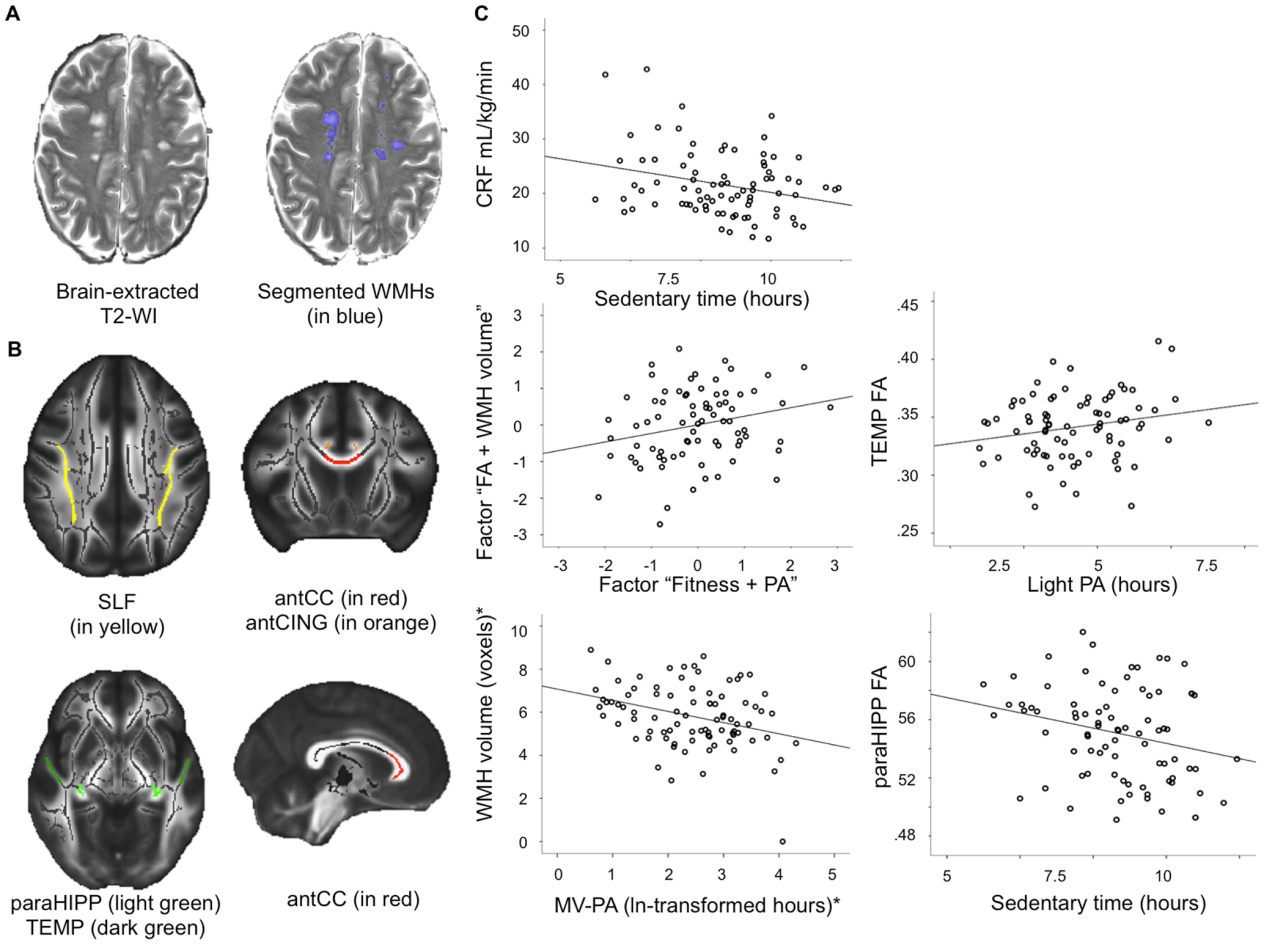
Illustration of WMH volume and FA analyses. A: An example of segmentation of WMHs on a T2-weighted image. B: Regions of interest for DTI analysis. Mean WM skeleton overlaid on FMRIB58_FA mean FA image. Anterior corpus callosum (antCC), anterior cingulum (antCING), superior longitudinal fasciculus (SLF), temporal lobe WM (TEMP), and parahippocampal WM (paraHIPP). C: Scatterplots showing the representative relationships between PA, CRF, and WM integrity. *Indicates that the variable was ln-transformed.

We estimated WMH volume on T2-weighted images using a semi-automated procedure based on FMRIB's Automated Segmentation Tool (FAST in FSL v.5.0.1, [39]. The procedure included: a) removal of the skull and non-brain tissue using the Brain Extraction Tool (BET) [40], b) segmentation of the image into three tissue types (grey and white matter, cerebrospinal fluid). All segmentation results were visually checked by AZB, c) manual masking of WMH regions within the cerebrospinal fluid segmentation (done by AZB, Figure 1-A). WMH volume was reported in voxel number classified in the above procedure as hyperintense, where voxel size was 0.86×0.86×4 mm^3^. The WMH volume was positively skewed (Kolmogorov-Smirnov test D(60)  = 0.28, p<0.05) and we performed a natural log-transformation of this variable for further analyses.

### DTI analysis

DTI allows inferences about WM microstructure in vivo by quantifying the magnitude and directionality of diffusion of water within a tissue [20]. Visual checks were performed on every volume of the raw data of every participant by AZB. In one dataset, one volume with the corresponding b-vectors and b-values was deleted from the dataset before processing due to artifact. Next, DTI data were processed using the FSL Diffusion Toolbox v.3.0 in a standard multistep procedure, including: a) motion and eddy current correction of the images and corresponding b-vectors, b) removal of the skull and non-brain tissue using the Brain Extraction Tool [40], and c) voxel-by-voxel calculation of the diffusion tensors. Using the diffusion tensor information, FA maps were computed using DTIFit within the FDT. All motion- and eddy-current outputs, as well as FA images were visually inspected.

We used tract-based spatial statistics (TBSS; 41,42], a toolbox within FSL v5.0.1 [43], to create a representation of main WM tracts common to all subjects (WM “skeleton”). This included: (1) nonlinear alignment of each participant's FA volume to the 1×1×1 mm^3^ standard Montreal Neurological Institute (MNI152) space via the FMRIB58_FA template using the FMRIB's Nonlinear Registration Tool (FNIRT, [44]), (2) calculation of the mean of all aligned FA images, (3) creation of the WM “skeleton” by perpendicular non-maximum-suppression of the mean FA image and setting the FA threshold to 0.25, and (4) perpendicular projection of the highest FA value (local center of the tract) onto the skeleton, separately for each subject. The outputs of all the above processing steps were carefully inspected by AZB.

To verify how the tracts of interest were related to CRF and PA, we extracted FA values from five regions: anterior corpus callosum (antCC; two most anterior sections of the corpus callosum as segmented after [45], anterior cingulum (antCING), superior longitudinal fasciculi (SLF), temporal lobe WM (TEMP), and WM of the medial temporal lobe (parahippocampal WM, paraHIPP; Figure 1-B; [46]. The regions were identified on the TBSS skeleton with the use of the DTI WM atlas to probe FA in the core parts of the selected tracts [47]. DTI data of five participants did not entirely cover the temporal lobes. Therefore, the above analyzes were done twice: once with all participants and once without the five participants (n = 78). The full sample analysis resulted in common mask not covering the temporal lobes and therefore those five subjects had missing data in those tracts.

It is important to note that the TBSS analysis used here inherently focuses on normal appearing WM, as the highest FA values perpendicular to the tract are being projected to the WM skeleton for further analysis. This means that there may be some bias for excluding voxels affected by WMH from FA analyses. We thus consider TBSS approach most suitable for the current study as it maximizes the independence of FA measures from WMH, in addition to circumventing the inter-subject anatomical variability in older populations.

An exploratory analysis relating CRF with FA across the whole WM skeleton was carried out in randomize, a voxel-wise permutation-based (5000 permutations) inference (Nichols and Holmes, 2002), with the threshold-free cluster enhancement option, and controlling for age and gender.

### Statistical analyses

To test the first study hypothesis, we performed a dimensionality reduction by running two separate principal component analyses (PCA) with varimax rotation. The first model included the four variables: CRF, sedentary time, light PA, and MV-PA. The second model included the WM health indices: five FA values and WMH volume. Both analyses yielded a one-component model and therefore represented “CRF and PA” and “WM health”, respectively. Importantly, a PCA analysis replacing the five FA values with global FA measure (mean FA over the entire WM skeleton) yielded the same outcome, confirming that the FA in the five WM regions are representative for the overall WM health.

All statistical analyses were performed using SPSS (v.16, SPSS Inc., Chicago, IL, USA). We used partial 2-tailed correlations (controlling for chronological age and gender) and multiple step-wise linear regressions to investigate the relationships among CRF, PA, WMH volume, and FA. The p values in these post-hoc analyses were not corrected for multiple comparisons.

The raw CRF, PA, WMH volume, and FA values is made available in Data S1.

### Ethics statement

A University of Illinois Institutional Review Board approved the study, and written informed consent was obtained from all participants and the study was performed in accordance with the 1964 Declaration of Helsinki. Participants received financial reimbursement.

## Results

### PA, CRF, and WM integrity: age- and gender-related trends

We summarized the descriptive statistics of main variables in our study and their correlations with chronological age in Table 1. There were no gender differences except for CRF (higher in males, p =  .001). CRF in males, MV-PA, and FA (except for temporal lobe WM) decreased with age, while WMH volume increased with age. The WMH volume and FA values are comparable with previous reports for healthy adults in this age range [22], [48]. Given age and gender differences in key variables, we included age and gender as covariates in further analyses.

CRF was positively related to light and MV-PA, and negatively related to sedentary behavior (all controlled for age and gender, Table 2; Figure 1-C). These results are important in showing that, in this sample of older low-active adults, all levels of PA as well as sedentary behavior were moderately related to CRF. We also investigated how daily wear time (valid hours), to a large extent reflecting waking hours, was related to different PA levels. As expected, we found that all levels of PA were positively related to valid hours, but not CRF (Table 2). In particular, sedentary hours accounted for most of the variability in the wearing time among our participants.

**Table 2:** Partial correlations between CRF and levels of PA.

	1. Valid hours	2. Sedentary	3. Light PA	4. MV-PA	5. CRF
1. Valid hours	1	**pr = .53**	**pr = .45**	**pr = .22**	pr = .18
		p< .001	p< .001	p = .041	p = .106
		df = 82	df = 82	df = 82	df = 79
2. Sedentary		1	**pr = −.48**	**pr = −.27**	**pr = −.36**
			p< .001	p = .013	p = .001
			df = 82	df = 82	df = 79
3. Light PA		-	1	**pr = .35**	**pr = .43**
				p = .001	p< .001
				df = 82	df = 79
4. MV-PA		-	-	1	**pr = .55**
					p< .001
					df = 79
5. CRF		-	-	-	1

All correlations controlled for age and gender. pr denotes partial correlation. CRF data is in units of mL/kg/min, Sedentary, Light, and MV-PA are expressed in hours (ln-transformed for MV-PA, winsorized for valid hours).

Finally, we examined the relationships between WMH volume and FA in the five WM regions. We found no significant relationship between WMH volume and FA (controlled for age and gender). This confirmatory analysis showed that in the five regions, WMH and DTI are not redundant measures of WM health.

### Greater CRF and PA are associated with better WM health

Dimensionality reduction using PCA separately on WM and CRF and PA measures yielded single factors for “CRF and PA” and “FA and WMH volume”. In other words, participants with greater “CRF and PA” factor scores had higher CRF, showed more light and MV-PA, and less sedentary behavior. Participants with higher WM health factor score had higher FA in all five regions and lower WMH volume. As predicted, we found a positive association between “CRF and PA” and “FA and WMH volume” (r =  .24, p =  .037, n = 77), which was a prerequisite for the following post-hoc analyses (Figure 1-C).

### Relationships of PA with WM health (FA and WMH volume)

MV-PA (pr = −.31 p =  .004, df = 82), but not sedentary or light daily PA was related to WMH volume (all controlled for age and gender; Figure 1-C). A multiple hierarchical regression showed that MV-PA was associated with WMH volume in addition to variance explained by age, gender, and CRF (Model 1 in Table 3).

**Table 3:** Regression models predicting WM integrity.

Dependent variables	Predictors	*R^2^ change*	*F change*	*df*	p-value F change
1. WMH volume (voxels)	Age, gender	.15	7.13	80/2	.**001**
	CRF	.02	2.19	79/1	.143
	MV-PA	.50	5.06	78/1	.**027**
2. Temporal WM	Age, gender	.08	3.10	75/2	**.051**
	CRF	.04	3.20	74/1	**.078**
	Light PA	.02	2.07	73/1	**.155**
3. Parahippocampal FA	Age, gender	.14	6.10	75/2	**.004**
	CRF	.002	0.16	74/1	.689
	Sedentary time	.08	7.03	73/1	.**010**
4. Parahippocampal FA	Age, gender	.15	7.08	78/2	.**001**
	MV-PA	.001	.09	77/1	.764
	Sedentary time	.05	4.55	76/1	**.036**

CRF data is in units of mL/kg/min, Sedentary and MV-PA are expressed in hours (ln-transformed for MV-PA).

Greater light PA (pr =  .25, p =  .026, df = 77) was associated with higher FA in the temporal lobe WM (Figure 1-C). A multiple hierarchical regression showed that light PA was not associated with temporal lobe FA in addition to variance explained by age, gender, and CRF (Model 2 in Table 3).

Sedentary behavior was inversely related to FA in the parahippocampal WM. That is, participants who spent more time on sedentary behavior had lower FA adjacent to the hippocampus (pr = −.24, p =  .035, df = 77; controlled for age and gender; Figure 1-C). The negative relationship between sedentary behavior and temporal lobe FA (pr = −.21, p =  .066, df = 77) was at trend level. Again, as sedentary behavior was negatively associated with CRF, we examined whether the effect of sedentariness on parahippocampal FA was independent of CRF in a hierarchical multiple regression. We found that sedentary behavior explained a significant amount of variance in parahippocampal FA, independently of variance explained by age, gender, and CRF (Model 3 in Table 3). The results of all of the above analyses were unchanged if valid hours were included as covariates in addition to age, gender, and CRF. Only the partial correlation between sedentary time and the parahippocampal WM FA was at a trend level after controlling for valid hours in addition to age and gender (pr = −.20, p =  .076, df = 76). This was expected, given that the variance in valid hours was mainly associated with variance in sedentary time and, to a lesser extent, to light and MV-PA. Together, these results suggest that daily valid PA measurement hours, likely reflecting waking hours, are to some extent related to the PA counts, but do not account for the relationships between PA and WM health.

Finally, the mechanisms linking sedentary behavior to brain integrity may differ from those related to MV-PA [7]. Indeed, a hierarchical multiple regression showed that sedentary behavior explained a significant amount of variance in parahippocampal FA, independently of variance explained by age, gender, and MV-PA (Model 4 in Table 3).

Figure 1-C shows scatterplots of the representative relationships between CRF, PA, and WM integrity.

### Relationships of CRF with WM health (FA and WMH volume)

To revisit the findings from previous studies [23]–[25] we tested the relationship between CRF and FA three prefrontal WM regions in our low-active and low-fit sample. We found no significant associations in the ROI-based analysis as well as whole-brain exploratory TBSS analysis. There was no significant relationship between CRF and WMH volume (pr = −.16, p =  .143, df = 79).

We found a positive association only between temporal lobe FA and CRF; however, after controlling for age and gender it remained at trend level (pr =  .20, p =  .078, df = 74).

## Discussion

We studied the relationships among CRF, PA, and WM health in a sample of 88 healthy low-active older adults. We found that “CRF and PA”, measured as a factor combining CRF and three levels of PA, was positively associated with WM health, represented by a factor “FA and WMH volume”. Post hoc analyses showed that this general result was driven by several WM-PA relationships. Specifically, higher MV-PA was associated with lower WMH volume, independent of age, gender, and CRF. Additionally, less time spent in sedentariness was linked with higher FA in the parahippocampal WM, and this association was independent of age, gender, and MV-PA. Our findings suggest that different levels of PA, although related, are associated with different aspects of WM integrity in a region-specific way. Here we focus on the specific post-hoc results and discuss how different mechanisms related to physical activity and sedentariness, such as cerebrovascular health, neurotrophic factors, or glucose and lipid metabolism, could underlie the observed relationships in specific brain regions.

### PA is related to WMH volume

We found that higher MV-PA was related to lower volume of WMH. We propose that the mechanism underlying this association is the beneficial effect of the MV-PA on the cerebrovascular system.

Aging is associated with stiffening and dilation of aorta and proximal elastic arteries due to accumulating “wear and tear” of their elastic and muscular components. This results in hypertension and impaired cushioning function of the arterial system, i.e. the ability to change the pulsatile flow in arteries to steady flow in the capillaries. This impaired cushioning increases vascular wall tension in arterioles and capillaries, particularly those of the brain [49]. This causes mechanical damage to blood vessel, arteriosclerotic changes, inflammation, as well as alterations in mechanosensitive gene expression, leading to vascular remodeling and oxidative stress [49], [50].

These age-related vascular changes are said to be counteracted by mainly moderate PA, captured as MV-PA by accelerometry. The effects of MV-PA include reduction in arterial stiffness and blood pressure [51], preserved arterial elasticity, blood flow, and reduced formation of arteriosclerotic lesions [52]. Clearly, these age-related vascular changes, which are modifiable by PA, overlap with the factors involved in the pathogenesis of WMH (permanent vessel dilation, damage to the vascular wall, disruption of the blood-brain-barrier, leakage of fluid from the blood vessel lumen to the surrounding tissue [17], [53]. Indeed, the association between hypertension and higher risk of WMH is well documented in large samples of older adults [54], [55]. However, the current study is first to report that link between lifestyle behavior relevant for cardiovascular health and WMH burden. To directly investigate how cerebrovascular factors relate to WM health, we are currently analyzing the relationships between cerebral arterial elasticity and WMH volume in another aging sample. We also speculate that in our relatively low-active and low-fit but healthy sample MV-PA may be a more sensitive correlate of WMH and FA than CRF, as VO_2_max reflects not only cardiovascular health, but also reflects body composition, genetic factors, and muscle metabolic rate. An ongoing exercise intervention study will shed more light on the discrepancy between CRF and MV-PA measures in relation to WM health.

### Time spent on light PA is related to temporal lobe WM integrity

Light PA was positively related to FA in the temporal lobe WM. Our results are novel in showing that not only exercise aimed to increase CRF, but also light PA may be beneficial for the temporal lobe during aging. It is worth noting, however, that in our low-fit sample, greater daily light PA (and not only MV-PA) was also associated with greater CRF, and light PA no longer significantly predicted temporal FA after accounting for CRF. Therefore, we speculate that the effect of PA on temporal WM may be related to mechanisms related to CRF.

The beneficial effects of CRF and exercise on temporal lobe structures are well known in both animals and humans [56]. Specifically, in older adults, aerobic exercise interventions aimed to increase CRF are related to increased gray matter density in the lateral temporal cortex [57]. A recent study showed that improvement in CRF due to aerobic exercise training correlated with an increase in FA of temporal and frontal lobes in older adults [58].

Aerobic exercise upregulates the expression and release of important neuronal growth factors such as brain-derived neurotrophic factor (BDNF) [31], [58]–[60] and insulin-like growth factor I (IGF-1; a potent survival factor for neurons and oligodendrocytes) [61]. Therefore, structural and functional brain changes related to improved oxidative capacity have been largely attributed to the action of neurotrophins. For example, an aerobic walking intervention in older adults resulted in increased temporal lobe functional connectivity, associated with increased BDNF, IGF-1, and vascular endothelial growth factor serum levels [58].

BDNF supports survival and growth of many neuronal subtypes and is a mediator of synaptic efficacy and use-dependent plasticity [62]. Still, the effects of BDNF on WM are poorly understood. Therefore the link between CRF, neurotrophin upregulation, and temporal WM integrity in healthy humans is less straightforward. Healthy adult Val/Val homozygotes of the BDNF Val^66^Met polymorphism (increased levels of activity-dependent release) had decreased FA compared to Met-allele carriers [63], [64], and lower structural brain connectivity [65]. Therefore, while for grey matter the met66 allele is almost invariably protective, BDNF seems to be a “punitive” signal in axonal pruning, i.e. higher BDNF secretion from stimulated axons results in its higher binding to p75 neurotrophin receptor on less active axons, which leads to neurite elimination [66] BDNF has neuroprotective role of on WM in clinical studies [67], [68] and plays a role in normal axonal pruning and maintenance observed in animals [66], [69]. Therefore, further research combining various MRI modalities to serum levels of neurotrophins is necessary to understand their role in mature as well as aging WM, and the link between light PA and temporal lobe WM.

It is also possible that the positive effects of light PA on temporal lobe WM integrity are mediated by changes in cerebrovascular supply factors related to CRF, reducing the oxygenative stress in the tissue [12]. Aerobic training correlates with increased capillary density in brain parenchyma [70] and higher number and integrity of small blood vessels as measured by MRI angiography [71]. Together, these changes may lead to increased ratio of blood vessels to brain volume [72] and more efficient oxygen delivery.

An alternative, although not mutually exclusive, explanation for the observed association between PA and WM integrity (both FA and WMH) is that individuals with higher WMH burden or decreases in FA are at more risk of impaired mobility [73], gait and balance dysfunction [74]–[79], and physical disability [80]–[82]. This could lead to their lower mobility and engagement in PA. Still, a study comparing athletes with non-exercising controls indicated that life-long exercise is associated with reduced WMH and age-related decline in FA [25]. We will test these alternative hypotheses in an ongoing randomized control study with exercise intervention.

In sum, our results suggest that not only structured exercise, but also light PA may be associated with greater WM microstructural integrity in temporal regions, known to be prone to age-related changes and sensitive to CRF-related benefits. Future analyses linking temporal WM FA to cognitive measures, local structure, and neural activity will allow assessing the functional relevance of the reported associations.

### Sedentary behavior may relate to WM via different mechanisms than PA

We found that older adults who spent more time in sedentary behavior had lower FA in the parahippocampal WM. Importantly, more sedentary behavior was related not only to less light PA, but also to less MV-PA, and lower CRF. Therefore, more sedentary older individuals were less exposed to beneficial effects of light and MV-PA, but also more prone to deleterious effects of sedentariness, such as increased metabolic risk [83], [84]. The association between sedentariness and parahippocampal WM, however, was independent from MV-PA. This is in line with recent hypotheses that sedentariness may act via different mechanisms than MV-PA, and may even offset the benefits of MV-PA [7], [85]. Specifically, both acute and chronic sedentariness is related to reduced activity of lipoprotein lipase activity [86], which facilitates uptake of fatty acids into muscle and adipose tissue. This causes increased plasma levels of triglycerides, total cholesterol, and decreased levels of high-density lipoprotein cholesterol [84], [87]. In addition, sedentary behavior is related to insulin resistance and increased glucose plasma levels [84].

Physiological differences between sedentary behavior and lack of MV-PA [7], [85] may partly explain the specificity of sedentary behavior for parahippocampal WM integrity. For instance, insulin receptors are highly expressed in the hippocampus and play a modulatory role in learning and memory processing [88]. Both insulin deficiency and resistance impairs hippocampus-dependent memory, long-term potentiation along the perforant pathway, and adult neurogenesis in rats [89]. Insulin resistance in humans is associated with similar cognitive deficits as in rodents [90]. Similarly, elevation in serum cholesterol change hippocampus lipid metabolism and induce oxygenative stress in rats [91]. As hyperlipidemia often presages insulin resistance, we speculate that sedentariness-induced changes in plasma lipids and glucose may have negative effect on hippocampus in low-active older adults, even in the absence of diagnosed diabetes. Deleterious changes in medial temporal lobe structures should be tightly linked to WM tracts connecting these regions. Little is known, however, about mechanisms linking plasma lipids and glucose levels to WM integrity, i.e. myelin turnover and repair, oligodendrocyte viability, axonal transport and integrity. Clearly, more research is needed to understand how sedentary behavior physiology interacts with the integrity of WM. Ideally, future studies will combine PA measures, structural and functional MRI with blood metabolite assays.

Finally, we observed no relationships between CRF with WMH volume or FA in the corpus callosum, the cingulum bundle, or superior longitudinal fasciculi, as reported previously by [23]–[25]. The earlier studies may have limited generalizability due to size (n≤26) and characteristics of the sample: [23] sample consisted of 26% superiorly fit individuals according to ACSM's Guidelines for Exercise Testing and Prescription (V0_2_max >42.7 for men in 7^th^ decade of life), 53% participants in [24] had a history of minimum 10 years of exercise 3 hours/week and mean V0_2_max  = 38, while half of [25] sample included Masters athletes. These effects observed in small samples including superiorly fit older individuals might not be reproducible in larger, low-active aging samples with narrower range of CRF, like ours. The causality of the relationships is also speculative, as some genetic or environmental factors, unrelated to PA, may have predisposed Masters athletes for high performance in aerobic sports and may be also linked to WM properties.

### Conclusions

We explored the associations of objective measures of CRF and PA with measures of WM health in older low-fit and low-active adults. We showed that higher levels of MV-PA were linked to lower WMH volume. We propose that MV-PA allows keeping WMHs in check via cerebrovascular mechanisms, such as preserving higher blood vessel elasticity and wall integrity. We found an association between light PA and WM integrity in the temporal lobe while sedentary behavior was related to lower FA in the parahippocampal WM. We speculate that these associations are related to neurotrophic, cerebrovascular, lipid and insulin metabolic mechanisms related to PA or lack thereof. Together, our findings suggest that PA is associated with WM health in aging in an intensity- and region-specific manner. Our results are optimistic in showing that modifiable lifestyle factors such as increasing PA and reducing sedentariness may be beneficial for brain health. We are currently assessing in a randomized exercise intervention trial whether increases in CRF and changes in PA behavior may induce changes in WM health over shorter periods of time.

We conclude that PA and CRF are related but not equivalent in their relationships with WM health in aging. Specifically, the effects are WM measure- and region-specific, showing the importance of using multiple, objective, and independent measures to assess fitness, physical activity and sedentariness, and brain integrity. Our findings pave the way for testing more targeted hypotheses linking bodily fitness and WM integrity in longitudinal designs, with improved public health recommendations on PA being the longer-term outcome of such studies.

## Supporting Information

Data S1 **The file contains the raw demographic, PA, CRF, DTI, and WMH data of the 103 participants.**
(XLSX)

## References

[1] Kramer AF, Hahn S, Cohen NJ, Banich MT, McAuley E, et al. (1999) Ageing, fitness and neurocognitive function. 10.1038/22682 10440369

[2] Hillman CH, Erickson KI, Kramer AF (2008) Be smart, exercise your heart: exercise effects on brain and cognition. Nat Rev Neurosci 9: 58–65. 18094706 10.1038/nrn2298

[3] Madden DJ, Bennett IJ, Burzynska A, Potter GG, Chen N-K, et al (2012) Diffusion tensor imaging of cerebral white matter integrity in cognitive aging. Biochim Biophys Acta 1822: 386–400. 21871957 10.1016/j.bbadis.2011.08.003 PMC3241892

[4] Raz N, Rodrigue KM (2006) Differential aging of the brain: patterns, cognitive correlates and modifiers. Neurosci Biobehav Rev 30: 730–748. 16919333 10.1016/j.neubiorev.2006.07.001 PMC6601348

[5] Freedson PS, Melanson E, Sirard J (1998) Calibration of the Computer Science and Applications, Inc. accelerometer. Med Sci Sports Exerc 30: 777–781. 9588623 10.1097/00005768-199805000-00021

[6] Craft LL, Zderic TW, Gapstur SM, Vaniterson EH, Thomas DM, et al (2012) Evidence that women meeting physical activity guidelines do not sit less: an observational inclinometry study. Int J Behav Nutr Phys Act 9: 122. 23034100 10.1186/1479-5868-9-122 PMC3490758

[7] Voss MW, Carr LJ, Clark R, Weng T (2014) Revenge of the “sit” II: Does lifestyle impact neuronal and cognitive health through distinct mechanisms associated with sedentary behavior and physical activity? Ment Health Phys Act.

[8] Middleton LE, Manini TM, Simonsick EM, Harris TB, Barnes DE, et al (2011) Activity energy expenditure and incident cognitive impairment in older adults. Arch Intern Med 171: 1251–1257. 21771893 10.1001/archinternmed.2011.277 PMC3923462

[9] Donahoo WT, Levine JA, Melanson EL (2004) Variability in energy expenditure and its components. Curr Opin Clin Nutr Metab Care 7: 599–605. 15534426 10.1097/00075197-200411000-00003

[10] Dunstan DW, Howard B, Healy GN, Owen N (2012) Too much sitting–a health hazard. Diabetes Res Clin Pract 97: 368–376. 22682948 10.1016/j.diabres.2012.05.020

[11] Helgerud J, Høydal K, Wang E, Karlsen T, Berg P, et al (2007) Aerobic high-intensity intervals improve VO2max more than moderate training. Med Sci Sports Exerc 39: 665–671. 17414804 10.1249/mss.0b013e3180304570

[12] Bassett DR, Howley ET (2000) Limiting factors for maximum oxygen uptake and determinants of endurance performance. Med Sci Sports Exerc 32: 70–84. 10647532 10.1097/00005768-200001000-00012

[13] Spina RJ, Ogawa T, Kohrt WM, Martin WH, Holloszy JO, et al (1993) Differences in cardiovascular adaptations to endurance exercise training between older men and women. J Appl Physiol 75: 849–855. 8226490 10.1152/jappl.1993.75.2.849

[14] Hoppeler H, Howald H, Conley K, Lindstedt SL, Claassen H, et al (1985) Endurance training in humans: aerobic capacity and structure of skeletal muscle. J Appl Physiol 59: 320–327. 4030584 10.1152/jappl.1985.59.2.320

[15] Peter I, Papandonatos GD, Belalcazar LM, Yang Y, Erar B, et al (2014) Genetic modifiers of cardiorespiratory fitness response to lifestyle intervention. Med Sci Sports Exerc 46: 302–311. 23899896 10.1249/MSS.0b013e3182a66155 PMC4055466

[16] Baloh RW, Vinters HV (1995) White matter lesions and disequilibrium in older people. II. Clinicopathologic correlation. Arch Neurol 52: 975–981. 7575225 10.1001/archneur.1995.00540340067014

[17] Pantoni L (2002) Pathophysiology of age-related cerebral white matter changes. Cerebrovasc Dis 13 Suppl 2 7–10. 10.1159/000049143 11901236

[18] Schmidt R, Enzinger C, Ropele S, Schmidt H, Fazekas F (2003) Progression of cerebral white matter lesions: 6-year results of the Austrian Stroke Prevention Study. Lancet 361: 2046–2048. 12814718 10.1016/s0140-6736(03)13616-1

[19] Basser PJ (n.d.) Inferring microstructural features and the physiological state of tissues from diffusion-weighted images. NMR Biomed 8: 333–344. 8739270 10.1002/nbm.1940080707

[20] Beaulieu C (n.d.) The basis of anisotropic water diffusion in the nervous system - a technical review. NMR Biomed 15: 435–455. 12489094 10.1002/nbm.782

[21] Bennett IJ, Madden DJ, Vaidya CJ, Howard DV, Howard JH (2010) Age-related differences in multiple measures of white matter integrity: A diffusion tensor imaging study of healthy aging. Hum Brain Mapp 31: 378–390. 19662658 10.1002/hbm.20872 PMC2826569

[22] Burzynska AZ, Preuschhof C, Bäckman L, Nyberg L, Li S-C, et al (2010) Age-related differences in white matter microstructure: region-specific patterns of diffusivity. Neuroimage 49: 2104–2112. 19782758 10.1016/j.neuroimage.2009.09.041

[23] Johnson NF, Kim C, Clasey JL, Bailey A, Gold BT (2012) Cardiorespiratory fitness is positively correlated with cerebral white matter integrity in healthy seniors. Neuroimage 59: 1514–1523. 21875674 10.1016/j.neuroimage.2011.08.032 PMC3230672

[24] Marks BL, Katz LM, Styner M, Smith JK (2011) Aerobic fitness and obesity: relationship to cerebral white matter integrity in the brain of active and sedentary older adults. Br J Sports Med 45: 1208–1215. 20558529 10.1136/bjsm.2009.068114

[25] Tseng BY, Gundapuneedi T, Khan MA, Diaz-Arrastia R, Levine BD, et al (2013) White matter integrity in physically fit older adults. Neuroimage 82: 510–516. 23769914 10.1016/j.neuroimage.2013.06.011 PMC3759589

[26] Voss MW, Heo S, Prakash RS, Erickson KI, Alves H, et al. (2012) The influence of aerobic fitness on cerebral white matter integrity and cognitive function in older adults: Results of a one-year exercise intervention. Hum Brain Mapp. 10.1002/hbm.22119 PMC4096122 22674729

[27] De Leeuw FE, de Groot JC, Achten E, Oudkerk M, Ramos LM, et al (2001) Prevalence of cerebral white matter lesions in elderly people: a population based magnetic resonance imaging study. The Rotterdam Scan Study. J Neurol Neurosurg Psychiatry 70: 9–14. 11118240 10.1136/jnnp.70.1.9 PMC1763449

[28] McAuley E, Szabo AN, Mailey EL, Erickson KI, Voss M, et al (2011) Non-Exercise Estimated Cardiorespiratory Fitness: Associations with Brain Structure, Cognition, and Memory Complaints in Older Adults. Ment Health Phys Act 4: 5–11. 21808657 10.1016/j.mhpa.2011.01.001 PMC3146052

[29] Szabo AN, McAuley E, Erickson KI, Voss M, Prakash RS, et al (2011) Cardiorespiratory fitness, hippocampal volume, and frequency of forgetting in older adults. Neuropsychology 25: 545–553. 21500917 10.1037/a0022733 PMC3140615

[30] Voss MW, Erickson KI, Prakash RS, Chaddock L, Malkowski E, et al (2010) Functional connectivity: a source of variance in the association between cardiorespiratory fitness and cognition? Neuropsychologia 48: 1394–1406. 20079755 10.1016/j.neuropsychologia.2010.01.005 PMC3708614

[31] Erickson KI, Voss MW, Prakash RS, Basak C, Szabo A, et al (2011) Exercise training increases size of hippocampus and improves memory. Proc Natl Acad Sci U S A 108: 3017–3022. 21282661 10.1073/pnas.1015950108 PMC3041121

[32] Erickson KI, Prakash RS, Voss MW, Chaddock L, Hu L, et al (2009) Aerobic fitness is associated with hippocampal volume in elderly humans. Hippocampus 19: 1030–1039. 19123237 10.1002/hipo.20547 PMC3072565

[33] Voss MW, Heo S, Prakash RS, Erickson KI, Alves H, et al (2013) The influence of aerobic fitness on cerebral white matter integrity and cognitive function in older adults: results of a one-year exercise intervention. Hum Brain Mapp 34: 2972–2985. 22674729 10.1002/hbm.22119 PMC4096122

[34] Vernooij MW, de Groot M, van der Lugt A, Ikram MA, Krestin GP, et al (2008) White matter atrophy and lesion formation explain the loss of structural integrity of white matter in aging. Neuroimage 43: 470–477. 18755279 10.1016/j.neuroimage.2008.07.052

[35] Haskell W, Lee I-M, Pate R, Powell K, Blair S, et al (2007) Physical Activity and Public Health: Updated Recommendation for Adults From the American College of Sports Medicine and the American Heart Association. Circulation 116: 1081–1093. 17671237 10.1161/CIRCULATIONAHA.107.185649

[36] Hart TL, McClain JJ, Tudor-Locke C (2011) Controlled and free-living evaluation of objective measures of sedentary and active behaviors. J Phys Act Health 8: 848–857. 21832301 10.1123/jpah.8.6.848

[37] Troiano RP, Berrigan D, Dodd KW, Mâsse LC, Tilert T, et al (2008) Physical activity in the United States measured by accelerometer. Med Sci Sports Exerc 40: 181–188. 18091006 10.1249/mss.0b013e31815a51b3

[38] Reese TG, Heid O, Weisskoff RM, Wedeen VJ (2003) Reduction of eddy-current-induced distortion in diffusion MRI using a twice-refocused spin echo. Magn Reson Med 49: 177–182. 12509835 10.1002/mrm.10308

[39] Zhang Y, Brady M, Smith S (2001) Segmentation of brain MR images through a hidden Markov random field model and the expectation-maximization algorithm. IEEE Trans Med Imaging 20: 45–57. 11293691 10.1109/42.906424

[40] Smith SM (2002) Fast robust automated brain extraction. Hum Brain Mapp 17: 143–155. 12391568 10.1002/hbm.10062 PMC6871816

[41] Smith SM, Jenkinson M, Johansen-Berg H, Rueckert D, Nichols TE, et al (2006) Tract-based spatial statistics: voxelwise analysis of multi-subject diffusion data. Neuroimage 31: 1487–1505. 16624579 10.1016/j.neuroimage.2006.02.024

[42] Smith SM, Johansen-Berg H, Jenkinson M, Rueckert D, Nichols TE, et al (2007) Acquisition and voxelwise analysis of multi-subject diffusion data with tract-based spatial statistics. Nat Protoc 2: 499–503. 17406613 10.1038/nprot.2007.45

[43] Smith SM, Jenkinson M, Woolrich MW, Beckmann CF, Behrens TEJ, et al (2004) Advances in functional and structural MR image analysis and implementation as FSL. Neuroimage 23 Suppl 1 S208–19. 15501092 10.1016/j.neuroimage.2004.07.051

[44] Rueckert D, Sonoda LI, Hayes C, Hill DL, Leach MO, et al (1999) Nonrigid registration using free-form deformations: application to breast MR images. IEEE Trans Med Imaging 18: 712–721. 10534053 10.1109/42.796284

[45] Hofer S, Frahm J (2006) Topography of the human corpus callosum revisited–comprehensive fiber tractography using diffusion tensor magnetic resonance imaging. Neuroimage 32: 989–994. 16854598 10.1016/j.neuroimage.2006.05.044

[46] Burzynska AZ, Garrett DD, Preuschhof C, Nagel IE, Li S-C, et al (2013) A scaffold for efficiency in the human brain. J Neurosci 33: 17150–17159. 24155318 10.1523/JNEUROSCI.1426-13.2013 PMC6618437

[47] Mori S, Wakana S, Nagae-Poetscher LM, Van Zijl PCM (2005) MRI Atlas of Human White Matter. Elsevier, editor Elsevier.

[48] Bhagat YA, Beaulieu C (2004) Diffusion anisotropy in subcortical white matter and cortical gray matter: changes with aging and the role of CSF-suppression. J Magn Reson Imaging 20: 216–227. 15269946 10.1002/jmri.20102

[49] O'Rourke MF, Hashimoto J (2007) Mechanical factors in arterial aging: a clinical perspective. J Am Coll Cardiol 50: 1–13. 17601538 10.1016/j.jacc.2006.12.050

[50] Ungvari Z, Kaley G, de Cabo R, Sonntag WE, Csiszar A (2010) Mechanisms of vascular aging: new perspectives. J Gerontol A Biol Sci Med Sci 65: 1028–1041. 20576649 10.1093/gerona/glq113 PMC2950814

[51] McDonnell BJ, Maki-Petaja KM, Munnery M, Yasmin, Wilkinson IB, et al (2013) Habitual exercise and blood pressure: age dependency and underlying mechanisms. Am J Hypertens 26: 334–341. 23382483 10.1093/ajh/hps055

[52] Vaitkevicius PV, Fleg JL, Engel JH, O'Connor FC, Wright JG, et al (1993) Effects of age and aerobic capacity on arterial stiffness in healthy adults. Circulation 88: 1456–1462. 8403292 10.1161/01.cir.88.4.1456

[53] Schmidt R, Petrovic K, Ropele S, Enzinger C, Fazekas F (2007) Progression of leukoaraiosis and cognition. Stroke 38: 2619–2625. 17673724 10.1161/STROKEAHA.107.489112

[54] Longstreth WT, Manolio TA, Arnold A, Burke GL, Bryan N, et al (1996) Clinical correlates of white matter findings on cranial magnetic resonance imaging of 3301 elderly people. The Cardiovascular Health Study. Stroke 27: 1274–1282. 8711786 10.1161/01.str.27.8.1274

[55] Van Dijk EJ, Breteler MMB, Schmidt R, Berger K, Nilsson L-G, et al (2004) The association between blood pressure, hypertension, and cerebral white matter lesions: cardiovascular determinants of dementia study. Hypertension 44: 625–630. 15466662 10.1161/01.HYP.0000145857.98904.20

[56] Voss MW, Vivar C, Kramer AF, van Praag H (2013) Bridging animal and human models of exercise-induced brain plasticity. Trends Cogn Sci 17: 525–544. 24029446 10.1016/j.tics.2013.08.001 PMC4565723

[57] Colcombe SJ, Erickson KI, Scalf PE, Kim JS, Prakash R, et al (2006) Aerobic exercise training increases brain volume in aging humans. J Gerontol A Biol Sci Med Sci 61: 1166–1170. 17167157 10.1093/gerona/61.11.1166

[58] Voss MW, Erickson KI, Prakash RS, Chaddock L, Kim JS, et al (2013) Neurobiological markers of exercise-related brain plasticity in older adults. Brain Behav Immun 28: 90–99. 23123199 10.1016/j.bbi.2012.10.021 PMC3544982

[59] Rasmussen P, Brassard P, Adser H, Pedersen M V, Leick L, et al (2009) Evidence for a release of brain-derived neurotrophic factor from the brain during exercise. Exp Physiol 94: 1062–1069. 19666694 10.1113/expphysiol.2009.048512

[60] Zoladz JA, Pilc A, Majerczak J, Grandys M, Zapart-Bukowska J, et al (2008) Endurance training increases plasma brain-derived neurotrophic factor concentration in young healthy men. J Physiol Pharmacol 59 Suppl 7 119–132. 19258661

[61] Carro E, Nuñez A, Busiguina S, Torres-Aleman I (2000) Circulating insulin-like growth factor I mediates effects of exercise on the brain. J Neurosci 20: 2926–2933. 10751445 10.1523/JNEUROSCI.20-08-02926.2000 PMC6772191

[62] Cotman CW, Berchtold NC (2002) Exercise: A behavioral intervention to enhance brain health and plasticity. Trends Neurosci 25: 295–301. 12086747 10.1016/s0166-2236(02)02143-4

[63] Chiang M-C, Barysheva M, Toga AW, Medland SE, Hansell NK, et al (2011) BDNF gene effects on brain circuitry replicated in 455 twins. Neuroimage 55: 448–454. 21195196 10.1016/j.neuroimage.2010.12.053 PMC3192852

[64] Tost H, Alam T, Geramita M, Rebsch C, Kolachana B, et al (2013) Effects of the BDNF Val66Met polymorphism on white matter microstructure in healthy adults. Neuropsychopharmacology 38: 525–532. 23132269 10.1038/npp.2012.214 PMC3547204

[65] Ziegler E, Foret A, Mascetti L, Muto V, Le Bourdiec-Shaffii A, et al (2013) Altered white matter architecture in BDNF met carriers. PLoS One 8: e69290. 23935975 10.1371/journal.pone.0069290 PMC3729843

[66] Cao L, Dhilla A, Mukai J, Blazeski R, Lodovichi C, et al (2007) Genetic modulation of BDNF signaling affects the outcome of axonal competition in vivo. Curr Biol 17: 911–921. 17493809 10.1016/j.cub.2007.04.040 PMC2175069

[67] Husson I, Rangon C-M, Lelièvre V, Bemelmans A-P, Sachs P, et al (2005) BDNF-induced white matter neuroprotection and stage-dependent neuronal survival following a neonatal excitotoxic challenge. Cereb Cortex 15: 250–261. 15269108 10.1093/cercor/bhh127

[68] Weinstock-Guttman B, Zivadinov R, Tamaño-Blanco M, Abdelrahman N, Badgett D, et al (2007) Immune cell BDNF secretion is associated with white matter volume in multiple sclerosis. J Neuroimmunol 188: 167–174. 17602759 10.1016/j.jneuroim.2007.06.003

[69] Singh KK, Park KJ, Hong EJ, Kramer BM, Greenberg ME, et al (2008) Developmental axon pruning mediated by BDNF-p75NTR-dependent axon degeneration. Nat Neurosci 11: 649–658. 18382462 10.1038/nn.2114

[70] Black JE, Isaacs KR, Anderson BJ, Alcantara AA, Greenough WT (1990) Learning causes synaptogenesis, whereas motor activity causes angiogenesis, in cerebellar cortex of adult rats. Proc Natl Acad Sci U S A 87: 5568–5572. 1695380 10.1073/pnas.87.14.5568 PMC54366

[71] Bullitt E, Rahman FN, Smith JK, Kim E, Zeng D, et al (2009) The effect of exercise on the cerebral vasculature of healthy aged subjects as visualized by MR angiography. AJNR Am J Neuroradiol 30: 1857–1863. 19589885 10.3174/ajnr.A1695 PMC7051270

[72] Thomas C, Baker CI (2013) Teaching an adult brain new tricks: a critical review of evidence for training-dependent structural plasticity in humans. Neuroimage 73: 225–236. 22484409 10.1016/j.neuroimage.2012.03.069

[73] Wakefield DB, Moscufo N, Guttmann CR, Kuchel GA, Kaplan RF, et al (2010) White matter hyperintensities predict functional decline in voiding, mobility, and cognition in older adults. J Am Geriatr Soc 58: 275–281. 20374403 10.1111/j.1532-5415.2009.02699.x PMC3764600

[74] Whitman GT, Tang Y, Lin A, Baloh RW, Tang T (2001) A prospective study of cerebral white matter abnormalities in older people with gait dysfunction. Neurology 57: 990–994. 11571322 10.1212/wnl.57.6.990

[75] Starr JM, Leaper SA, Murray AD, Lemmon HA, Staff RT, et al (2003) Brain white matter lesions detected by magnetic resonance [correction of resosnance] imaging are associated with balance and gait speed. J Neurol Neurosurg Psychiatry 74: 94–98. 12486275 10.1136/jnnp.74.1.94 PMC1738198

[76] Srikanth V, Beare R, Blizzard L, Phan T, Stapleton J, et al (2009) Cerebral white matter lesions, gait, and the risk of incident falls: a prospective population-based study. Stroke 40: 175–180. 18927448 10.1161/STROKEAHA.108.524355

[77] Srikanth V, Phan TG, Chen J, Beare R, Stapleton JM, et al (2010) The location of white matter lesions and gait—a voxel-based study. Ann Neurol 67: 265–269. 20225293 10.1002/ana.21826

[78] Bhadelia RA, Price LL, Tedesco KL, Scott T, Qiu WQ, et al (2009) Diffusion tensor imaging, white matter lesions, the corpus callosum, and gait in the elderly. Stroke 40: 3816–3820. 19797696 10.1161/STROKEAHA.109.564765 PMC3401013

[79] Van Impe A, Coxon JP, Goble DJ, Doumas M, Swinnen SP (2012) White matter fractional anisotropy predicts balance performance in older adults. Neurobiol Aging 33: 1900–1912. 21872363 10.1016/j.neurobiolaging.2011.06.013

[80] Sachdev PS, Wen W, Christensen H, Jorm AF (2005) White matter hyperintensities are related to physical disability and poor motor function. J Neurol Neurosurg Psychiatry 76: 362–367. 15716527 10.1136/jnnp.2004.042945 PMC1739526

[81] Gouw AA, Van der Flier WM, van Straaten ECW, Barkhof F, Ferro JM, et al (2006) Simple versus complex assessment of white matter hyperintensities in relation to physical performance and cognition: the LADIS study. J Neurol 253: 1189–1196. 16998647 10.1007/s00415-006-0193-5

[82] Zheng JJJ, Delbaere K, Close JCT, Sachdev P, Wen W, et al (2012) White matter hyperintensities are an independent predictor of physical decline in community-dwelling older people. Gerontology 58: 398–406. 22614074 10.1159/000337815

[83] Demiot C, Dignat-George F, Fortrat J-O, Sabatier F, Gharib C, et al (2007) WISE 2005: chronic bed rest impairs microcirculatory endothelium in women. Am J Physiol Heart Circ Physiol 293: H3159–64. 17766475 10.1152/ajpheart.00591.2007

[84] Hamburg NM, McMackin CJ, Huang AL, Shenouda SM, Widlansky ME, et al (2007) Physical inactivity rapidly induces insulin resistance and microvascular dysfunction in healthy volunteers. Arterioscler Thromb Vasc Biol 27: 2650–2656. 17932315 10.1161/ATVBAHA.107.153288 PMC2596308

[85] Tremblay MS, Colley RC, Saunders TJ, Healy GN, Owen N (2010) Physiological and health implications of a sedentary lifestyle. Appl Physiol Nutr Metab 35: 725–740. 21164543 10.1139/H10-079

[86] Hamilton MT, Hamilton DG, Zderic TW (2004) Exercise physiology versus inactivity physiology: an essential concept for understanding lipoprotein lipase regulation. Exerc Sport Sci Rev 32: 161–166. 15604935 10.1097/00003677-200410000-00007 PMC4312662

[87] Yanagibori R, Kondo K, Suzuki Y, Kawakubo K, Iwamoto T, et al (1998) Effect of 20 days' bed rest on the reverse cholesterol transport system in healthy young subjects. J Intern Med 243: 307–312. 9627145 10.1046/j.1365-2796.1998.00303.x

[88] Wickelgren I (1998) Tracking insulin to the mind. Science 280: 517–519. 9575095 10.1126/science.280.5363.517

[89] Stranahan AM, Arumugam TV, Cutler RG, Lee K, Egan JM, et al (2008) Diabetes impairs hippocampal function through glucocorticoid-mediated effects on new and mature neurons. Nat Neurosci 11: 309–317. 18278039 10.1038/nn2055 PMC2927988

[90] Greenwood CE, Winocur G (2005) High-fat diets, insulin resistance and declining cognitive function. Neurobiol Aging 26 Suppl 1 42–45. 16257476 10.1016/j.neurobiolaging.2005.08.017

[91] Stranahan AM, Cutler RG, Button C, Telljohann R, Mattson MP (2011) Diet-induced elevations in serum cholesterol are associated with alterations in hippocampal lipid metabolism and increased oxidative stress. J Neurochem 118: 611–615. 21682722 10.1111/j.1471-4159.2011.07351.x PMC3137681

